# Left Vagus Stimulation Modulates Contralateral Subthalamic β Power Improving the Gait in Parkinson’s Disease

**DOI:** 10.1002/mds.29690

**Published:** 2023-12-18

**Authors:** Massimo Marano, Gaia Anzini, Luca Saltarocchi, Riccardo Ricciuti, Fioravante Capone, Huiling Tan, Flavie Torrecillos, Jacopo Lanzone, Vincenzo Di Lazzaro

**Affiliations:** 1 Unit of Neurology, Neurophysiology, Neurobiology and Psychiatry, Department of Medicine and Surgery, Università Campus Bio-Medico di Roma, Roma, Italy; 2 Fondazione Policlinico Universitario Campus Bio-Medico, Roma, Italy; 3 Neurosurgery Unit, Ospedale Belcolle, ASL di Viterbo, Viterbo, Italy; 4 Medical Research Council Brain Network Dynamics Unit, Nuffield Department of Clinical Neurosciences, University of Oxford, Oxford, United Kingdom; 5 Department of the Neurorehabilitation, Istituti Clinici Scientifici Maugeri IRCCS, Milano Institute, Milan, Italy

**Keywords:** non-invasive vagus nerve stimulation (VNS), β band, freezing of gait, transauricular vagus stimulation, deep brain stimulation (DBS)

## Abstract

**Background:**

Transcutaneous vagus nerve stimulation (VNS) showed early evidence of efficacy for the gait treatment of Parkinson’s disease (PD).

**Objectives:**

Providing data on neurophysiological and clinical effects of transauricular VNS (taVNS).

**Methods:**

Ten patients with recording deep brain stimulation (DBS) have been enrolled in a within participant design pilot study, double-blind crossover sham-controlled trial of taVNS. Subthalamic local field potentials (β band power), Unified Parkinson’s Disease Rating Scales (UPDRS), and a digital timed-up-and-go test (TUG) were measured and compared with real versus sham taVNS during medication-off/DBS-OFF condition.

**Results:**

The left taVNS induced a reduction of the total β power in the contralateral (ie, right) subthalamic nucleus and an improvement of TUG time, speed, and variability. The taVNS-induced β reduction correlated with the improvement of gait speed. No major clinical changes were observed at UPDRS.

**Conclusions:**

taVNS is a promising strategy for the management of PD gait, deserving prospective trials of chronic neuromodulation. © 2023 The Authors. *Movement Disorders* published by Wiley Periodicals LLC on behalf of International Parkinson and Movement Disorder Society.

## Introduction

Gait dysfunction is a main determinant of quality of life in patients with Parkinson’s disease (PD). PD symptoms, such as bradykinesia and rigidity, are associated with the overexpression of pathological brain rhythms in the β band within the subthalamic nucleus (STN).^1^ Both levodopa and STN deep brain stimulation (DBS) can disrupt such an aberrant synchronization.^1,2^ However, gait troubles are pitfalls for pharmacological and DBS therapies and severe gait issues may contraindicate surgery.^3^ Impaired walking abilities are caused by the degeneration of widespread networks, involving non-dopaminergic pathways.^4^ Nevertheless, the overexpression of β rhythms is still broadly associated to walking difficulties in PD.^5^ Because of its multimodal mechanism of action, transcutaneous vagus nerve stimulation (tVNS) has been recently proposed as a possible solution for PD axial signs (ie, gait, freezing).^6^ The acute administration of auricular or cervical tVNS induced a modest gait improvement at different PD stages with and without levodopa.^7,8^ More-over, the acute administration of cervical tVNS improved the β power with a possible cumulative effect.^9^ However, it is still not clear whether the VNS-induced improvement of gait in PD is modulated through the reduction of β oscillations, or whether it is because of changes in other activities. Here, the present study investigated the effect of auricular tVNS (taVNS) on the STN β activity and on the gait of PD patients in a double-blind sham-controlled study performed during their off condition.

## Methods

**FIG. 1:**
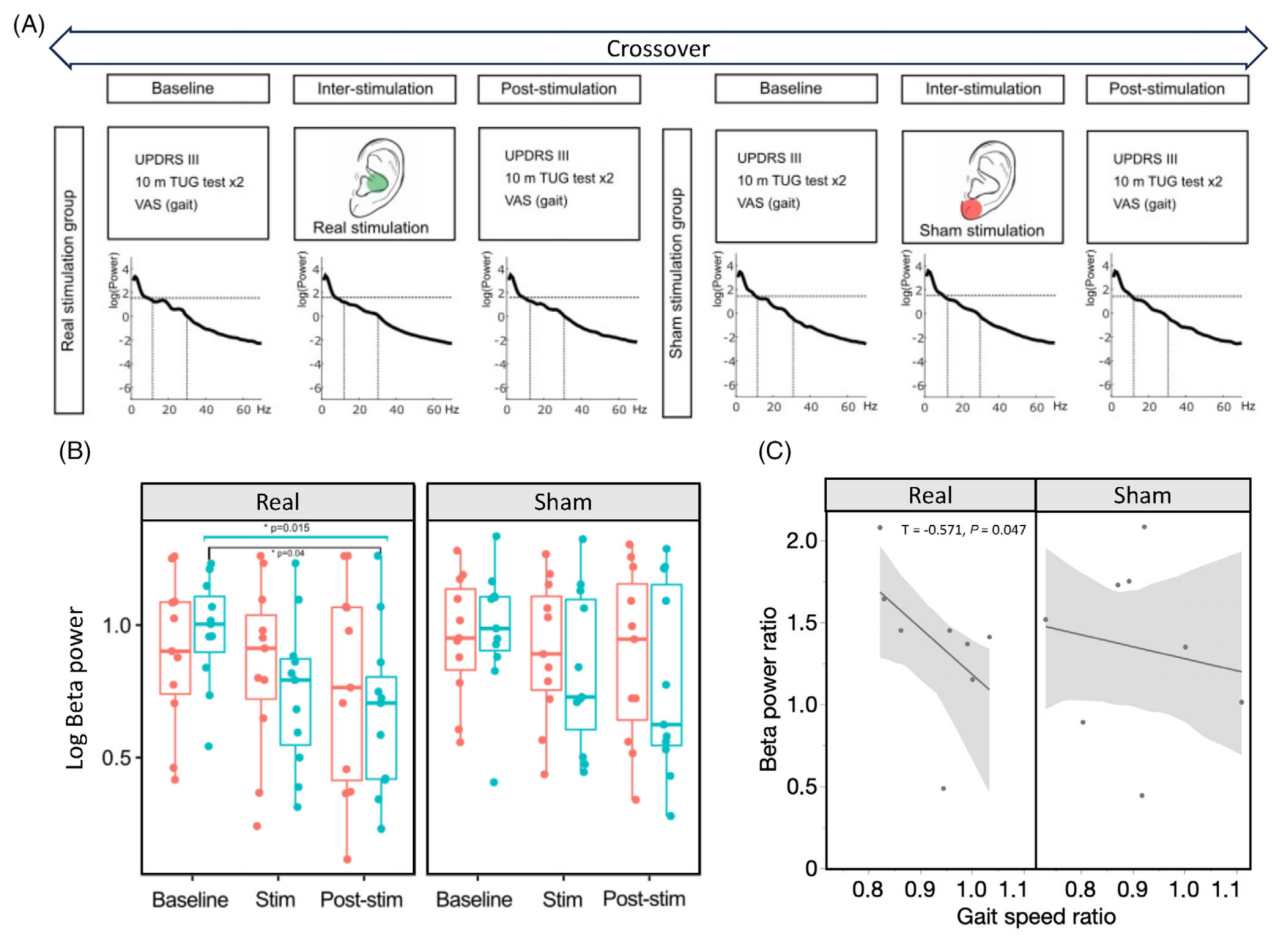
(A) Study design. The PSD of a patient is reported across time points and conditions. (B) Comparison between left (red) and right (blue) sub-thalamic nucleus β power. (C) Relationship between β power and speed ratio. [Color figure can be viewed at wileyonlinelibrary.com]

Ten PD patients with STN-DBS were enrolled. Enrollment criteria included: stable therapy for at least 1 month and STN-DBS (Percept™PC, Medtronic, PLM, MN, USA)^10^ for at least 2 months; ability to walk unassisted while on therapy; absence of contrain-dications for taVNS and of anticholinergic medications; and dementia. The study was approved by the ethical committee, in accordance with the Helsinki declaration. All participants gave their written informed consent. Data will be made available on request to the corresponding author. All patients were evaluated in the morning, after an overnight dopaminergic medication withdrawal (∼12 hours). On the day of the experiment, the DBS was turned off and the experiment was performed 1 hour after (MED-off/DBS-OFF). Each participant was tested with a real taVNS and a sham stimulation with the testing order randomized and the testing condition double blinded (Fig. 1). All subjects received four trains of stimulation (train duration 120 seconds, stimulation frequency 25 Hz, pulse duration 0.3 ms) with inter-train intervals of 60 seconds. Electrodes were placed in the left external acoustic meatus at the inner side of the tragus for real taVNS and attached to the left ear lobe for control stimulation as described else-where.^8^ All subjects received a baseline (V0) and a post-stimulation (V1) assessment with the Unified Parkinson’s Disease Rating Scales part III (UPDRS-III) and a single sensor digital gait analysis during a 10-m timed-up-and-go test (TUG) (www.mon4t.com). Participants’ blinding was veri-fied through a visual analogue scale (VAS) (Supporting Information S1 and S2).

Local field potentials (LFPs) have been recorded through the indefinite streaming of the Percept.^9^ Continuous LFPs signals were segmented in three periods as follows: baseline (60 seconds before the first VNS block), inter-stimulation (3 × 60 seconds), and post-stimulation (60 seconds after the last block). The channels with the best baseline β (13−30 Hz), representative of the somatomotor dorsolateral STN,^11^ were further analyzed to test their differences across time points and stimulation conditions in both average spectral power and frequency of the β peak. Difference between the average of the low (13−20 Hz) and high (20−30 Hz) β power was also explored.

Data have been reported as means (±standard deviations). Gait differences were tested through the Wilcoxon signed rank test for paired data with the false discovery rate correction. Differences across power spectral densities have been tested at different time-points and conditions with a permutation approach (Monte Carlo method) and a cluster-based correction.^12^ The β power obtained by the selected contacts was log transformed to approximate to a normal distribution and tested with repeated measures analysis of variance. Correlations have been investigated through the Kendall test. A *P* < 0.05 was adopted for statistical significance. Statistics have been performed through JMP 17.0 (SAS).

## Results

Our sample included 10 patients (two women) with an age of 59.2 ± 6.9 and a disease duration of 9.1 ± 2.3. The mean modified Hoehn and Yahr (MED-off/DBS-OFF) score and levodopa equivalent daily dose were 2.65 ± 0.6 and 340 ± 219 mg, respectively. Patients demonstrated to be unaware of the condition they received. There were no differences between the baseline values of the two visit days and of the sessions (Supporting Information S1 and S2).

The permutation analysis on the full PSD did not show any significantly different cluster for the tested time points (baseline vs. inter-stimulation; inter-stimulation vs. post-stimulation; baseline vs. post-stimulation), both for the sham and real groups. The β band analysis showed the presence of a progressive and significant reduction of the mean β power across time points. This was observed only over the right STN (contralateral to the side to which taVNS was applied) after the real stimulation (degrees of freedom, df = 10, F = 5.19, *P* = 0.015). However, a minor statistical trend was observed also over the left STN (df = 10, F = 3.03, *P* = 0.06). No significant changes were observed after the sham stimulation (Table 1). Such observation was further confirmed by the post hoc test, which found a significant difference between the right channels of the real condition in base-line versus post-stimulation β values (df = 30, T = 2.62, *P* = 0.013, adjusted-*P* = 0.040). The β-band power changes are illustrated in Fig. 1. We did not find signifi-cant differences in β peak frequency and in the average of low and high β power in the three test conditions of both groups (Supporting Information S1 and S2).

Eight subjects concluded the TUG task. Two participants dropped out the gait analysis because of the inability to walk and perform the task while in MED-off/DBS-OFF (n = 1) and because of the presence of corrupted data after visual inspection (n = 1). The mean performance of the TUGs was compared across visits and conditions. There was a significant improvement in the step variability ([Δ] = 0.06 *R*^2^), in the total time ([Δ] = 2.1 seconds) and in the walking speed ([Δ] = 0.08 m/s) between V0 and V1 only after the real taVNS (Table 1). Nevertheless, there were no significant UPDRS-III changes (total and axial sub-items).

To define the possible relationship between VNS-induced β power changes and the gait, we performed a correlation study between the pre-stimulation to post-stimulation ratios of β power, TUG time, walking speed, and step variability. The β power ratio was inversely related to the walking speed ratio (real stimulation, T = −0.571, *P* = 0.047) (Fig. 1).

## Discussion

The present study suggests that the acute administration of a few minutes of non-invasive VNS performed at the internal tragus of the left ear interacted with the right STN circuit and improved subclinical gait parameters of patients with PD, while not on dopaminergic therapies. The auricular branch of the vagus nerve (ABVN) is an afferent vagus branch and is accessible through the inner tragus, providing a door for the electric stimulation of the ascending vagus and of the central nervous system. ABVN fibers projects to the nucleus of the solitary tract and then to the locus coeruleus, activating the noradrenergic and the cholinergic systems. Finally, projections get to the cortex through the thalamus acting on GABAergic neurotransmission and improving markers of cortical excitability.^13,14^ Anatomical and functional connections between the vagus ascending network, the STN, and the striatum have been described.^15^ Nevertheless, its full functionality has been poorly investigated.

The VNS, whether invasive or non-invasive, interacts with brain rhythms. Indeed, through the putative interaction between the thalamus and the cortex, the VNS led to the desynchronization of networks, which were pathologically over expressed at lower frequencies (θ, α, and β), in other disease models.^16–19^ In a recent experiment, the left cervical tVNS reduced right and left STN low-β power.^9^ Here, the taVNS showed a prominent effect on the right side. This was possibly because of mechanisms of ascending system laterality or to the stimulation site (the cervical vagus system might recruit a larger neural substrate), to the neural degeneration, or to factors of aging and sex biology.^20–22^ The lack of taVNS related modulation on high and low β power is not surprising given their constitutional variability across subjects and phenotypes and the low sample size—a study on a large sample is warranted.

In our sample reported here, the taVNS effect on gait was not large enough to induce any clinically relevant change in the UPDRS III total and subscores. Modest gait changes occurred in the patients’ velocity during the TUG task, whereas a minor trend was observed at a parameter of gait variability—consistently with other VNS experiments.^7^ Step-to-step variability is a function of the cholinergic system^23^ and might be improved by strategies targeting that specific pathway (eg, cholinesterase inhibitors, pedunculopontine DBS).^24–26^ Given the unmet need of complementary treatments for non-dopaminergic symptoms, future trials investigating VNS effect on axial functions should be cautiously auspicated.

Our study has the main limitation of being a pilot trial, with a small sample size. Gait changes were insufficient to be clinically relevant at UPDRS-III, despite the β band improvement. However, even if the therapeutic usefulness of taVNS is still questionable, our results show early proofs of neural engagement.^27,28^ With the current methodology it is not possible to draw any further conclusion on the association between gait speed changes and β power. Indeed, the latter is an acknowledged biomarker of bradykinesia and rigidity and of their modulation through therapies,^29^ and its relationship with gait is controversial.^5^ β power and gait performances have a low-moderate association. The β band per se does not play a major role on gait, but stepping is associated to transient changes of STN LFP oscillatory states with the STN encoding gait phases.^30,31^ However, pathological gait patterns relate to more complex deep and cortical oscillatory behaviors.^32^ In the PD picture, the side matters.^33^ More specifically, recent evidence hypothesized the presence of a functional basal ganglia laterality, where the right hemisphere is dominant for the axial motor control. In line with this, Lizarraga et al^34^ observed that right side STN DBS contributed with a slightly greater improvement to the gait kinematics than left stimulation. Further experiments on the neurophysiological underpinnings of our findings are warranted, including extended stimulation trials of both hemispheres. Future clinical trials should consider the repositioning of existing devices as a chance to speed up the development of new therapeutic strategies for PD.

## Supplementary Material

Supplementary Materials

## Figures and Tables

**Table 1:** Difference across visits and conditions (sham stimulation; real taVNS) of clinical, gait parameters and STN β power

	Sham stimulation					Real taVNS			
Variable	V0	V1		*P*	V0	V1		*P*
UPDRS III (MED_off_/STIM_OFF_)	27.4 ± 17.5	27.5 ± 19.5		0.382	28.5 ± 19.7	29.4 ± 20.8		0.468
Stand time (s)	1.89 ± 0.14	1.93 ± 0.13		0.570	1.84 ± 0.13	1.89 ± 0.09		0.312
Rotation time (s)	1.97 ± 0.04	1.97 ± 0.07		0.921	1.94 ± 0.05	1.96 ± 0.05		0.484
Total time (s)	28.83 ± 4.64	26.90 ± 4.05		0.054	28.79 ± 5.48	26.63 ± 4.86		**0.007**
Steps (n)	25.5 ± 6.6	25.7 ± 5.4		0.796	26 ± 6	26.2 ± 5.4		1.000
Sway (m)	0.05 ± 0.01	0.04 ± 0.01		0.546	0.04 ± 0.01	0.04 ± 0.01		1.000
Walking speed (m/s)	1.01 ± 0.21	1.12 ± 0.23		0.109	1.09 ± 0.26	1.17 ± 0.28		**0.027**
Step variability (*R*^2^)	0.43 ± 0.09	0.43 ± 0.10		1.000	0.40 ± 0.14	0.47 ± 0.13		**0.035**
Stride length (m)	0.65 ± 0.15	0.66 ± 0.11		0.382	0.65 ± 0.13	0.66 ± 0.10		0.726
	Baseline	Inter-stim	Post-stim		Baseline	Inter-stim	Post-stim	
Right STN β power	2.72 ± 0.69	2.29 ± 0.68	2.28 ± 0.84	0.18	2.69 ± 0.62	2.26 ± 0.67	2.13 ± 0.75	**0.015**
Left STN β power	2.65 ± 0.59	2.64 ± 0.54	2.49 ± 0.8	0.11	2.53 ± 0.58	2.43 ± 0.72	2.26 ± 0.85	0.06

Statistical significance after correction for multiple comparisons in bold.

Abbreviations: taVNS, transauricular vagus nerve stimulation; STN, subthalamic nucleus; UPDRS-III, Unified Parkinson’s Disease Rating Scales part III; MED, medication.

## Data Availability

Data available on request from the authors.
