# Supplementary material for: Left Vagus Stimulation Modulates Contralateral Subthalamic β Power Improving the Gait in Parkinson’s Disease
Source: Mov Disord. Author manuscript; Available in PMC 2024 Apr 18. (PMC7615838; doi:10.1002/mds.29690)
Supplement: Supplementary Materials [file EMS195314-supplement-Supplementary_Materials.zip › mds29690-sup-0001-supinfo.docx]

**Supplementary methods**

**Stimulation protocol materials**

The stimulation protocol was performed through an electric stimulator (Digitimer DS7, Digitimer Ltd, UK) and two Ag–AgCl electrodes (5 mm in diameter with a distance between the cathode and anode of about 5 mm). The electrical stimulator was triggered by Signal software (version 5) and CED data acquisition interface (Cambridge Electronic Design, Cambridge, UK).

**LFPs recording protocol.**

Patients’ STN LFPs were recorded through the indefinite streaming modality of the Percept device at the baseline, during and after the stimulation of both conditions. Data coming from the device consisted of bipolar montages created between adjacent contacts (L01, L12, L23 - left STN and R01, R12, R23 - right STN ventral-to-dorsal order). LFPs were analyzed offline using custom-written scripts in MATLAB (2019a, Mathworks, Massachusetts, USA). Continuous LFPs signals were then segmented in three periods as follows: baseline (30s before the first VNS block), inter-stimulation (3 * 60s), and post-stimulation (30s after the last block). For each period, the power spectral densities (PSD) were estimated in consecutives 5s time-windows (pwelch method, 50% overlap) and averaged across all time-windows. The contacts used to perform the statistical analysis are reported in supplementary table 1 together with patients’ therapeutic contacts and lead reconstructions (SureTune4, Medtronic).

**Randomization and verification of the patient awareness about the stimulation condition**

Random allocation of patients for the first condition was performed by an independent researcher through random allocation cards using computer-generated random numbers. Four patients (40%) were randomized to start with the real condition. After each stimulation all subjects were asked to guess about their chance of being treated with a real stimulation on a VAS scale (VASdb; question “how much do you rate your confidence about having received a real treatment from 0 to 10”).

**Supplementary results**

**High versus Low beta band power.** In supplementary table 4 and supplementary figure 2 we show how real taVNS and sham stimulation affected the high (20-30 Hz) and low (13-20 Hz) beta power in the contact with the highest total beta power contralateral to the stimulation side in each patient (i.e., closest to the STN). We did not find statistically any statistically significant difference; however, the post-stimulation condition of the real taVNS group shows an apparent reduction in the low beta power variability deserving further investigation with larger samples of patients.

**Adverse events.** Adverse events were observed in 2 cases out of 10 reporting blurred vision (n=1) and neck pain (n=1) for a few hours (regressed spontaneously) after the real taVNS and the sham stimulation, respectively. Both were judged as mild and unrelated.

**Supplementary table 1.** Baseline patient disease features.

|  | | **Mean ± standard deviation** | **n (%)** |
| --- | --- | --- | --- |
| Modified Hoehn and Yahr (Medication OFF / DBS ON) | |  |  |
|  | 2 |  | 4 (40%) |
|  | 2.5 |  | 3 (40%) |
|  | 3 |  | 3 (40%) |
| Modified Hoehn and Yahr (Medication OFF / DBS OFF) | |  |  |
|  | 2 |  | 3 (30%) |
|  | 2.5 |  | 3 (30%) |
|  | 3 |  | 3 (30%) |
|  | 4 |  | 1 (10%) |
| UPDRS II total score | |  |  |
|  | Item “freezing” = 0 |  | 5 (50%) |
|  | Item “freezing” = 1 |  | 3 (30%) |
|  | Item “freezing” = 2 |  | 1 (10%) |
|  | Item “freezing” = 3 |  | 1 (10%) |
|  | Item “gait” = 1 |  | 8 (80%) |
|  | Item “gait” = 2 |  | 2 (20%) |
| UPDRS III (MED_OFF_/DBS_ON_) (real) | | 14.6 ± 9.8 |  |
| UPDRS III (MED_OFF_/DBS_ON_) (sham) | | 13.2 ± 7.8^ns^ |  |
| VASdb (real) | | 5.4 ± 1.7 |  |
| VASdb (sham) | | 5.7 ± 2.4^ns^ |  |

Levodopa equivalent daily dose (LEDD, mgs); Unified Parkinson’s Disease Rating Scale (UPDRS). UPDRS II item “freezing when walking”: none = 0; rare freezing when walking; may have hesitation = 1; occasional freezing when walking = 2; occasionally falls from freezing = 3; frequent falls from freezing = 4. UPDRS item “walking”: normal = 0; mild difficulty, may not swing arms or may tend to drag leg = 1; moderate difficulty, but requires little or no assistance = 2; severe disturbance of walking, requiring assistance = 3; cannot walk at all, even with assistance = 4; VAS for double blind assessment (VASdb). ^ns^statistics vs the baseline value of the real stim group have a p-value >0.05.

**Supplementary table 2.** Contacts and lead locations used for recordings and stimulation.

|  | Recording bipolar montage | Stimulating contacts | Lead location |
| --- | --- | --- | --- |
| Patient 1 | R ch 1-2;L ch 1-2 | R2, L2 | Suppl. figure 1A |
| Patient 2 | R ch 1-2;L ch 1-2 | R2, L12 | Suppl. figure 1B |
| Patient 3 | R ch 2-3;L ch 2-3 | R2 , L2 | Suppl. figure 1C |
| Patient 4 | R ch 1-2;L ch 1-2 | R2 , L2 | Suppl. figure 1D |
| Patient 5 | R ch 1-2;L ch 1-2 | R2, L2 | Suppl. figure 1E |
| Patient 6 | R ch 2-3;L ch 1-2 | R2, L1 | Suppl. figure 1F |
| Patient 7 | R ch 1-2;L ch 1-2 | R12, L12 | Suppl. figure 1G |
| Patient 8 | R ch 1-2;L ch 1-2 | R2, L2 | Suppl. figure 1H |
| Patient 9 | R ch 1-2;L ch 1-2 | R12, L12 | Suppl. figure 1I |
| Patient 10 | R ch 2-3;L ch 2-3 | R2, L2 | Suppl. figure 1L |

Ch, channel (contact); R, right lead, L, left lead

**Supplementary table 3.** Differences across baseline evaluations of first (V1) versus the second (V2) visit.

| **Variable** | **V1** | **V2** | **p-value** |
| --- | --- | --- | --- |
| **UPDRS III (**MED_OFF_/STIM_OFF_**)** | 23.6±12.6 | 23.7±4.6 | 0.964 |
| **Stand time (s)** | 1.84±0.13 | 1.89±0.14 | 0.371 |
| **Rotation time (s)** | 1.97 ± 0.06 | 1.94 ± 0.01 | 0.171 |
| **Total time (s)** | 28.7±4.66 | 28.8±5.47 | 0.916 |
| **Steps (n)** | 26.8±4.5 | 24.6±7.5 | 0.597 |
| **Sway (m)** | 0.04±0.01 | 0.05±0.01 | 0.344 |
| **Walking speed (m/s)** | 1.04±0.21 | 1.06±0.26 | 0.752 |
| **Step variability (R^2^)** | 0.40±0.11 | 0.43±0.13 | 0.674 |
| **Stride length (m)** | 0.62 ±0.08 | 0.69 ±0.17 | 0.528 |

*Statistically significant changes after correcting for multiple comparisons according to the false discovery rate or Benjamini–Hochberg procedure.

**Supplementary table 4.** Low vs High beta power analysis under sham and real taVNS conditions and timepoints.

|  | Sham | | |  |
| --- | --- | --- | --- | --- |
| Beta band power | Pre | Stim | Post | p-value |
| Low beta | 3±1.4 | 2.94±1.5 | 2.94±1.8 | ns |
| High beta | 1.50±0.68 | 1.44±0.65 | 1.30±0.65 |  |
|  | Real | | |  |
|  | Pre | Stim | Post | p-value |
| Low beta | 2.96±1.22 | 2.92±1.16 | 2.79±1.38 | ns |
| High beta | 1.5±0.7 | 1.53±0.74 | 1.34±0.62 |  |

**Supplementary figure 1.** Lead location reconstruction with SureTune 4 software (Medtronic) based on patients’ anatomy. Subthalamic nucleus in green, red nucleus in light red, substantia nigra in dark red.
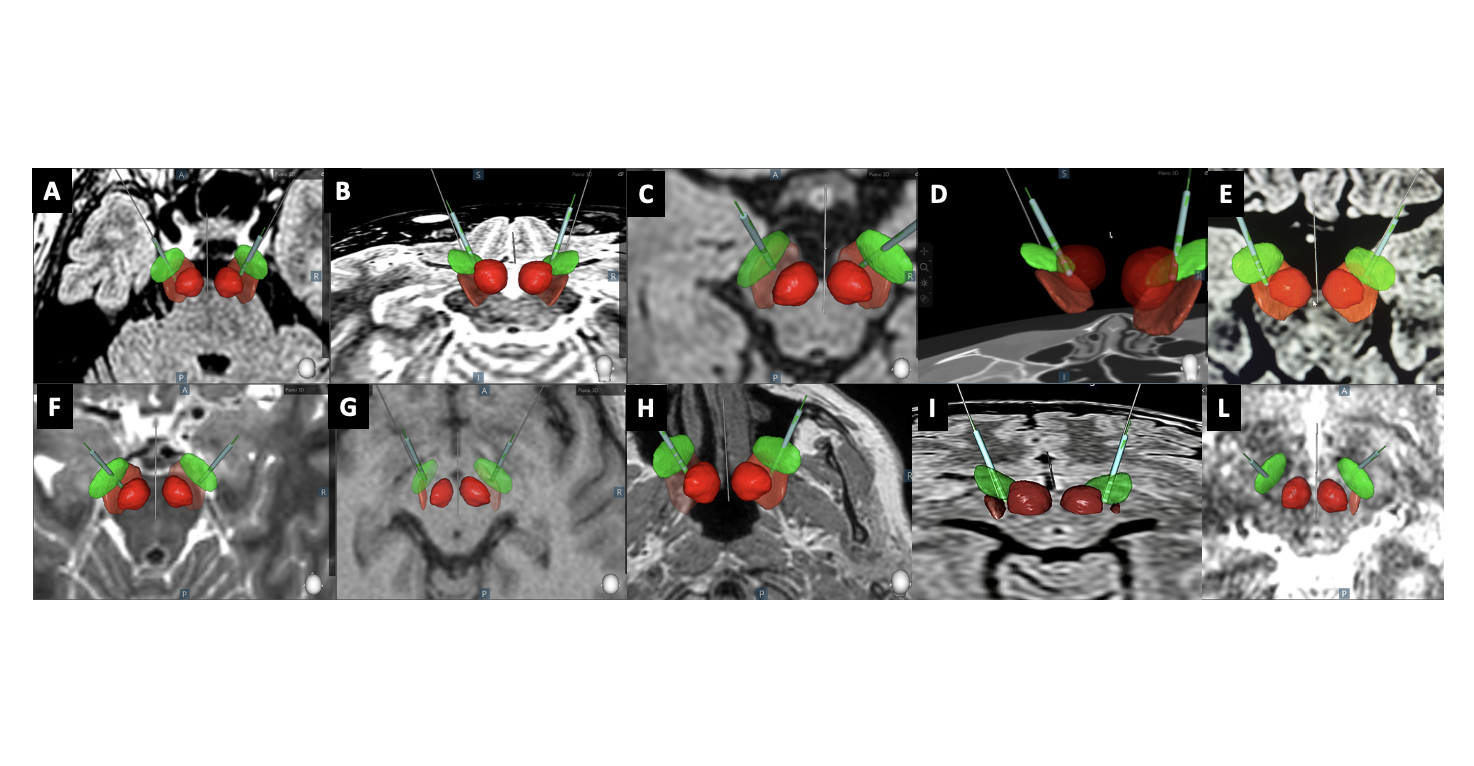


**Supplementary figure 2.** Distribution of low and high beta power across groups and timepoints.
